# The WNT Pathway Is Relevant for the BCR-ABL1-Independent Resistance in Chronic Myeloid Leukemia

**DOI:** 10.3389/fonc.2019.00532

**Published:** 2019-06-24

**Authors:** Susanna Grassi, Sara Palumbo, Veronica Mariotti, Diego Liberati, Francesca Guerrini, Elena Ciabatti, Serena Salehzadeh, Claudia Baratè, Serena Balducci, Federica Ricci, Gabriele Buda, Lorenzo Iovino, Francesco Mazziotta, Francesco Ghio, Giacomo Ercolano, Antonello Di Paolo, Antonella Cecchettini, Chiara Baldini, Letizia Mattii, Silvia Pellegrini, Mario Petrini, Sara Galimberti

**Affiliations:** ^1^Hematology Division, University of Pisa, Pisa, Italy; ^2^Department of Medical Biotechnologies, University of Siena, Siena, Italy; ^3^Department of Surgical, Medical and Molecular Pathology and Critical Care, University of Pisa, Pisa, Italy; ^4^Department of Clinical and Experimental Medicine, University of Pisa, Pisa, Italy; ^5^National Research Council of Italy, Milan, Italy; ^6^Department of Clinical and Experimental Medicine, Pharmacology Division, University of Pisa, Pisa, Italy; ^7^Department of Clinical and Experimental Medicine, Rheumatology Division, University of Pisa, Pisa, Italy

**Keywords:** WNT/β-catenin, PcGs, JAK/STAT, CML, BCR/ABL1-independent resistance, PCA

## Abstract

Notwithstanding the introduction of Tyrosine Kinase Inhibitors (TKIs) revolutionized the outcome of Chronic Myeloid Leukemia (CML), one third of patients still suspends treatment for failure response. Recent research demonstrated that several BCR/ABL1-independent mechanisms can sustain resistance, but the relationship between these mechanisms and the outcome has not yet been fully understood. This study was designed to evaluate in a “real-life” setting if a change of expression of several genes involved in the WNT/BETA-CATENIN, JAK-STAT, and POLYCOMB pathways might condition the outcome of CML patients receiving TKIs. Thus, the expression of 255 genes, related to the aforementioned pathways, was measured by quantitative PCR after 6 months of therapy and compared with levels observed at diagnosis in 11 CML patients, in order to find possible correlations with quality of response to treatment and event-free-survival (EFS). These results were then re-analyzed by the principal component method (PCA) for tempting to better cluster resistant cases. After 12 months of therapy, 6 patients achieved an optimal response and 5 were “resistant;” after application of both statistical methods, it was evident that in all pathways a significant overall up-regulation occurred, and that WNT was the pathway mostly responsible for the TKIs resistance. Indeed, 100% of patients with a “low” up-regulation of this pathway achieved an optimal response vs. 33% of those who showed a “high” gene over-expression (*p* = 0.016). Analogously, the 24-months EFS resulted significantly influenced by the degree of up-regulation of the WNT signaling: all patients with a “low” up-regulation were event-free vs. 33% of those who presented a “high” gene expression (*p* = 0.05). In particular, the PCA analysis confirmed the role of WNT pathway and showed that the most significantly up-regulated genes with negative prognostic value were DKK, WNT6, WISP1, and FZD8. In conclusion, our results sustain the need of a wide and multitasking approach in order to understand the resistance mechanisms in CML.

## Introduction

Chronic myeloid leukemia (CML) is a clonal myeloproliferative disorder originated from hematopoietic stem cell progenitors characterized by the presence of the Philadelphia chromosome (Ph) and the BCR/ABL1 fusion gene. The BCR/ABL1 oncoprotein is a constitutively active tyrosine kinase that promotes the activation of different transduction pathways involved in cell growth and differentiation (RAS, RAF, JUN, MYC, STAT, AKT), with the consequent transformation of the hematopoietic stem cell in a neoplastic clone. Ph'+ cells are genetically unstable and prone to have multiple heterogeneous genetic aberrations that could induce the transformation into acute leukemia, leading to the transformation from one chronic disease to an accelerated or a blastic phase (1).

A crucial event in this transition seems to be the acquisition of mutations in the kinase domain of the ABL1 gene, with the consequent resistance to the tyrosine kinase inhibitors (TKIs). Resistance, during therapy, is frequently linked to the inability of TKIs to arrange in the ATPase pocket of the fusion protein due to point mutations in the ATP-binding task. About 4% of failing cases presents ABL1 mutations, even at the low level (sub-clonal), detectable only by NGS whose sensitivity is higher than offered by the Sanger sequencing (2). The T315I mutation confers resistance to the TKIs imatinb, dasatinib, nilotinib, and bosutinib, whereas it is sensitive to ponatinib (3). Nevertheless, combination with other mutations (compound mutations), also ponatinib is ineffective, and other therapeutical approaches are necessary, such as asciminib (4).

For the majority of cases where the resistance to TKIs is not related to the ABL1 mutations different mechanisms have been already hypothesized:

1. The protective action exerted by the bone marrow niche, where hypoxia and glucose deprivation block the production of the fusion protein in the CML leukemic stem cell (LSC), with the consequent ineffective action of TKIs that cannot reach their target (5, 6);
2. An aberrant expression of some genes involved in the epigenetic control, such as those belonging to the Polycomb family; for example, over-expression of BMI1 resulted to be a negative prognostic molecular marker in terms of event-free survival (EFS) (7). The negative role of BMI1 oncogenic protein can be suggested by the fact that it is co-localized in the cytoplasm of LSC with the BCR-ABL1 P210 protein and with CD26 (8), the dipeptidil peptidase IV (DPPIV) that has been shown to represent a specific marker of CML progenitors (9, 10);
3. The hyper-activation of the JAK-STAT signaling. This pathway is involved in the transduction of signals mediated by cytokines, interferon and growth factors, with consequent support of neoplastic cell growth and invasion in many types of cancer. Additional implications in inflammation and immunity have been also recently attributed to this pathway, thus supporting the idea that it could sustain the CML LSC maintenance (11, 12): indeed, the persistent STAT3 activation seems to confer resistance to TKIs by controlling the LSC self-renewal and by favoring its hiding in the bone marrow niche. Inhibition of this pathway might represent a potential way to ameliorate the molecular response in warning or failed CML patients or to sustain deep molecular responses in cases tempting the discontinuation of therapy (13).
4. The WNT/Beta-catenin pathway, that is necessary to the self-renewal of normal cells, but whose deregulation causes leukemogenesis and progression in several neoplasias (14) seems to be relevant in CML also. After linking to its receptors, WNT ligand induces the inhibition of beta-catenin phosphorylation and its cytoplasmic accumulation, with consequent activation of transcription and expansion of the leukemic progenitors. LSCs have aberrant regulation of beta-catenin, and in a CML murine model BCR/ABL1 seems to stimulate beta-catenin through the phosphoinositide 3-kinase (PI3K/AKT) signaling, then increasing the leukemic progression (15).
5. Polymorphisms of several transmembrane drug transporters. They can be predictive of response or intolerance in CML patients: indeed, it has been demonstrated that single nucleotide polymorphisms (SNP) of hOCT1, ABCB1, and ABCG2 conditioned the imatinib pharmacokinetics and disposition whereas they did not impact on Nilotinib efficacy or toxicity (16–20). This could be relevant in the decision of which kind of TKI has to be prescribed in the first line.

In this complex scenario, we decided to investigate the expression levels of some genes reported to sustain the BCR-ABL1-independent resistance in CML: in particular, we focused on JAK-STAT, WNT/beta-catenin, and Polycomb pathways, evaluating their de-regulation after 6 months of treatment from diagnosis. In addition to the “classical” supervised statistical analysis, we adopted also The Principal Component Analysis (PCA) method, that allows an unsupervised clustering analysis, in order to detect some otherwise not easily identifiable correlation between different gene expression profiles.

## Patients and Methods

### Patients

Patients included in the study were a small part of those already enrolled in two our previous studies designed to investigate the role of polymorphisms during treatment with TKIs [16, 20].

All cases were observed at the Hematology Unit of the University of Pisa (Italy) and consecutively enrolled on the basis of their acceptance to sign an informed consent to donate the leftover of samples harvested for routine diagnostic tests for every further no-profit scientific purpose. The informed consent was previously approved by the Azienda Ospedaliero-Universitaria Pisana (AOUP) Ethical Committee.

Clinical characteristics of the enrolled patients are reported in Table 1.

**Table 1:** Clinical characteristics of the enrolled patients.

**Clinical features**	***n* (%)**
**Patients**	11
**Age (median/range)**	69 (55–76)
**Sex**	
M	7 (63.6)
F	4 (36.4)
**Risk score:**	
**Sokal**	
Low	4 (40)
Intermediate	3 (20)
High	4 (40)
**Eutos**	
Low	8 (72)
Intermediate	2 (18)
High	1 (10)
**Elts**	
Low	6 (55)
Intermediate	3 (27)
High	2 (18)
**TKI as I line**	
Imatinib	8 (73)
Dasatinib	1 (9)
Nilotinib	2 (18)

*M, male; F, female*.

### Methods

#### BCR/ABL1 Quantification

Buffy coats obtained from 10 to 20 mL of peripheral blood anti-coagulated with EDTA have been used for the extraction of total RNA by using the phenol/chlorophorm method. To remove contaminating phenol, RNAs were cleaned up using silica-membrane technology (RNeasy Kit, QIAGEN, Valencia, CA, USA), and to avoid DNA-contamination RNAs were treated with DNase (New England Biolabs, Ipswich, MA, USA).

Total RNA concentrations was evaluated by using a ND-1000 spectrophotometer (NanoDrop, Wilmington, DE, USA), and the RNA quality assessed by BioAnalyzer (Agilent, Santa Clara, CA, USA).

Total RNA was then reverted into cDNA by the Ipsogen® RT Kit (Ipsogen, Milan, Italy), according to the manufacturer's instructions.

The molecular response, assessed by quantitative PCR (qPCR) for both P210 fusion transcripts, was calculated after alignment of PCR results to the international scale, as previously described in the ELN guidelines (21) and according to the rules edited by the Italian molecular network LabNet. Patients were classified as “responders,” “warning,” or “failing” according to the ELN guidelines (22).

Considering the small number of cases involved in the present studies, for a more powerful statistical analysis, patients were re-classified as “optimal” (#1, #4, #5, #6, #7, #9) or “failing” (#2, #3, #8, #10, #11) including in this latest category even those “warning,” in accordance to BCR-ABL1/ABL1 levels measured at 12 months. The quality of responses was assessed after 3, 6, and 12 month of treatment, and scored as reported in Supplementary Table 4.

The patients that did not achieve clinical response at fixed time points had been screened for the ABL1 mutational status and they did not harbor ABL1 mutations.

#### Gene Expression Analysis

For measuring the expression of 255 genes from the WNT, JAK-STAT and Polycomb pathways the commercial PrimePCR^TM^ SYBR® Green assay plates (BioRad) were used in a Real Time PCR apparatus (CFX, Connect^TM^, BioRad), according to the manufacturer's instructions (for a detailed list of genes see Supplementary Tables 1–3).

We analyzed samples at diagnosis and after 6 months of therapy. Expression values were calculated by the Vandesompele method using four internal housekeeping genes (ABCTB GAPDH, HPRT, and TBP). Data have been also evaluated with the “Gene Study PrimePCR analysis” software and expressed as fold changes in respect of diagnosis.

#### Statistical Analysis

The statistical analysis of clinical outcome was performed by using chi-square test for the dicotomic variables or the ROC analysis in order to test a possible correlation between the value of gene expression de-regulation and quality of response. For the survival, the Kaplan-Meier method was employed. The SPSS version 25.0 software was used for the conventional above listed statistical analysis. For all tests, the level of significance was set at *p* ≤ 0.05.

The PCA analysis was carried out using the MatLab (*matrix laboratory*) software; MatLab is a multi-paradigm-numerical computing platform and matrix-based language developed by MathWorks. MatLab allows to analyze data, develop algorithms, create of user models, and applications, interfacing with programs written in other languages. In the study the PCAs were performed according to Liberati et al. (23).

## Results

### Clinical Outcome

All patients were diagnosed in chronic phase, their median age was 69 years (range, 56–79), 64% were men, and 36% women. Patients received imatinib (8), or second-generation TKIs (3) as first line of therapy and during the median follow-up of 24 months; 4 switched therapy, 3 for unsatisfactory response and one for grade-4 toxicity (Supplementary Table 5).

There was no correlation between type of therapy and clinical response, outcome or EFS, probably due to the small number of patients receiving second-generation TKIs enrolled in the study.

The early molecular response (BCR-ABL1/ABL1 ratio IS% <10% at 3 months) (EMR) was achieved by 70% of patients (8 cases); at 6 months of treatment one patient already failed response, two were warning and the remaining eight achieved an optimal response (BCR-ABL1/ABL1 ratio IS% <1%). At 12 months, 6 patients (54.5%) achieved an optimal response (BCR-ABL1/ABL1 ratio IS% <0.1%), whereas 2 were warning and 3 failed treatment (BCR-ABL1/ABL1 ratio IS% >1%).

At 18 and 24 months, the molecular responses were superimposable with those observed at month 12th (Supplementary Table 4).

The EFS was defined as the time elapsed from treatment beginning and the occurrence of one of the following events: failure at 12 months, drug discontinuation for any significant toxicity (WHO grade 4 or lower degree but persistent), progression, loss of complete cytogenetic response (CCyR), and/or of MR3 (BCR-ABL1/ABL1 ratio IS% <0.1%), or death for any cause.

Overall 24 months-EFS in our series was 55%; as expected, it was significantly influenced by the quality of the last molecular response (80% for optimal responders vs. 20% of failing cases; *p* = 0.018), by the EMR (75% for who achieved EMR vs. 0% for who not achieved EMR; *p* = 0.004), and by the permanent discontinuation of treatment for any cause (75% for responsive cases vs. 0% for the failing ones; *p* = 0.001%).

### Gene Expression Analysis

After 6 months of therapy a significant de-regulation of the majority of tested genes was observed: de-regulation was considered as significant if a fold change ≥2 (either for increased or decreased expression) (24) was detected (Table 2). To test whether the overall “expression burden” could play a prognostic role, the mean de-regulation value was calculated for each of the 3 considered pathways, and two categories were defined: (1) with “low” expression (when the overall number of de-regulated genes was lower than the respective mean value), and (2) with “high” expression (when the overall number of de-regulated genes was higher than the respective mean value). In all the 3 analyzed pathways an overall gene up-regulation was observed, especially for the JAK/STAT pathway (Table 3).

**Table 2:** Gene expression de-regulation of selected genes and function.

**Gene**	**Median fold-change**	**Optimal**	**Failure**	**Function**
DKK1	434.25	0.25	868.24	WNT signaling canonical via negative regulation, that acts by isolating the LRP6 co-receptor
WISP1	79.20	3.94	192.09	Wnt-induced secreted protein, anticancer activity, cell proliferation
FZD7	11.08	1.20	25.89	7-transmembrane domain protein that is receptor for Wnt signaling
MYC	5.62	2.02	11.02	Proto-oncogene encodes a nuclear phosphoprotein that plays a role in cell cycle progression, apoptosis and cellular transformation
WNT3	5.06	5.71	1.78	WNT canonical and WNT/Ca+2 non-canonical via
IL2RA	4.04	2.69	6.06	Receptors that bind and activate JAK proteins, immune response, cell cycle and apoptosis
IFNGR1	3.95	2.95	5.45	Receptors that bind and activate JAK proteins, immune response and cell growth
IRF1	3.49	4.47	2.02	INF regulatory factor 1 is a tumor suppressor gene and transcriptional activator of interferon-a/b
CEBPD	3.45	2.39	5.06	Gene induced by STAT3
STAT2	3.08	4.14	1.47	Tumor suppressor gene, anti-proliferative and pro-apoptotic effect
SIRT1	2.91	2.18	4.37	Polycomb Complex Interacting Genes
PIAS2	2.86	2.83	2.89	Transcriptional regulators of the JAK/STAT pathway
MLL5	2.61	2.36	3.13	Polycomb complex interacting genes, regulates transcription and differentiation
USP7	2.58	2.21	3.31	Polycomb complex interacting gene, recombination and DNA repair
LRP6	2.23	1.77	2.93	Function
PIAS1	2.23	1.88	2.75	Transcriptional regulators of the JAK/ STAT pathway, activatorof notch via
RBBP5	2.17	2.09	2.32	Tritorax core component, cell cycle and proliferation
PHC3	2.13	1.31	3.78	Polycomb complex core components, tumor progression
L3MBTL2	1.59	2.10	0.58	Polycomb complex interacting genes, pathogenesis of myeloid malignancies
IL4R	1.11	0.50	2.01	Receptors that bind and activate JAK protein via STAT6, immune response

*Median fold-change, median of the fold-change expression in all patients; Optimal, median of the fold-change expression only in optimal patients; Failure, median of the fold change expression in failing patients*.

**Table 3:** Number of de-regulated genes.

**Expression**	**Median**	**Average**	**Range**
**JAK-STAT**			
**Up** *n*° genes	45.5	46.3	(22–75)
**Down** *n*° genes	3.0	3.1	(0–9)
**Stable** *n*° genes	35.5	34.6	(9–53)
**WNT**			
**Up** *n*°genes	40.5	38.9	(27–46)
**Down** *n*°genes	12.5	14.0	(9–23)
**Stable** *n*°genes	24.0	23.8	(16–30)
**POLYCOMB**			
**Up** *n*°genes	32	33	(12–55)
**Down** *n*°genes	5	5	(1–10)
**Stable** *n*°genes	46	46	(10–69)

***Up**: >2 fold up-regulated genes; **Down**: >2 fold down-regulated genes; **Stable**: gene with <2 fold de-regulation*.

**Figure 1:**
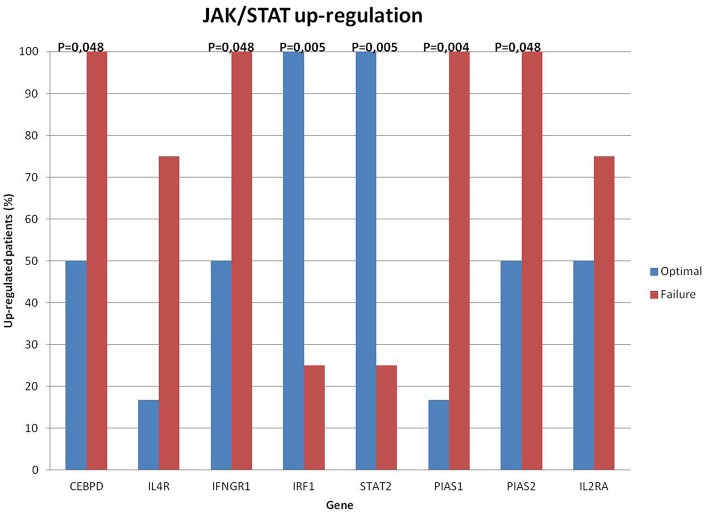
JAK/STAT significantly de-regulated genes. Optimal responders in blue columns and failing patients in red. Up-regulation of some JAK/STAT genes was significantly correlated to patients clinical response (*p* ≤ 0.05).

When we tested the correlation between the overall gene over-expression of the JAK/STAT pathway and the quality of response to TKIs, we observed that 80% of cases in optimal response belonged to the category with lower up-regulation; nevertheless, this correlation was not statistically significant (*p* = 0.189). Analogously, also the EFS was not significantly conditioned by the expression levels of the genes belonging to this pathway. Further, some de-regulated genes, when individually analyzed, showed a significantly correlation with clinical response and outcome (Figure 1). The 24 month-EFS was correlated to the up-regulation of: IRF1 (*p* = 0.008; 71% up-regulated vs. 0%), STAT 2: (*p* = 0.002; 71% up-regulated vs. 0%), PIAS1 (*p* = 0.033; 80% down–regulated vs. 20%).

**Figure 2:**
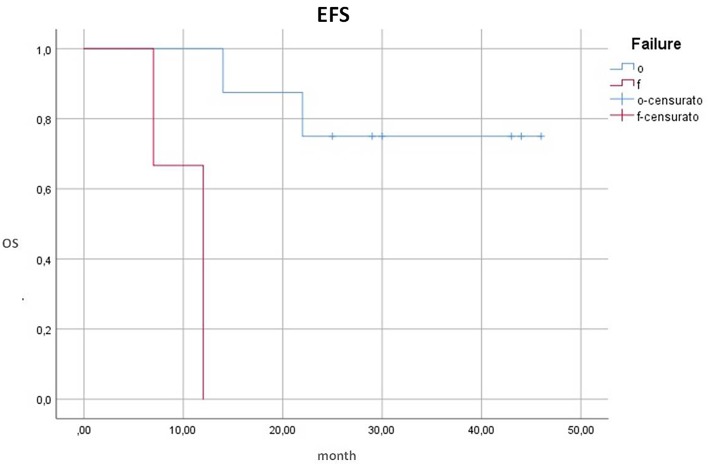
EFS and up-regulation of WNT pathway. EFS, event-free survival; OS, overall survival; WNT up, overexpression of WNT genes; 38, low expression; 39, high expression. 100% of patients with a “low” up-regulation were event-free vs. 33% of those who presented a “high” up-regulation (*p* = 0.05).

Then, we tested if the gene expression could be correlated to the quality of response in the remaining pathways: we observed that 100% of patients with a lower up-regulation (activation) of the WNT pathway achieved an optimal response vs. only 33% of subjects who showed a higher up-regulation of the WNT pathway (*p* = 0.016). Analogously, also the 24 months EFS resulted significantly influenced by the degree of up-regulation of this pathway: all patients with a “low” up-regulation were event-free vs. 33% of those who presented a “high” up-regulation (*p* = 0.05) (Figure 2).

**Figure 3:**
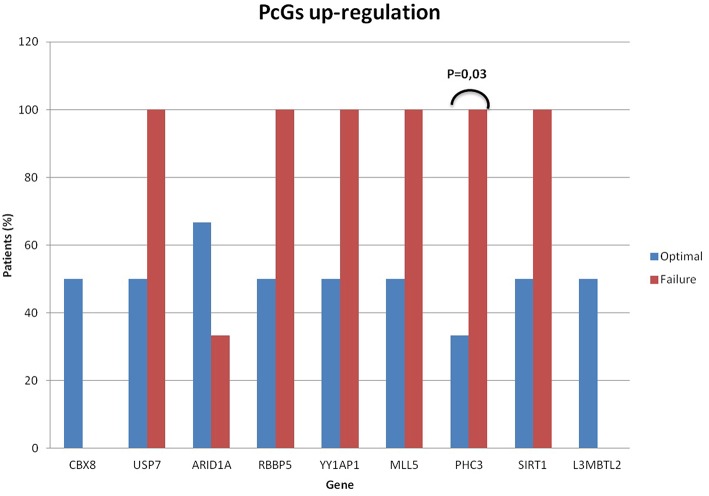
PcGs significantly de-regulation. Optimal responders in blue columns and failing patients in red. De-regulation of some polycomb genes was related to the clinical response, with a significantly up-regulation of PHC3 and worse response to TKIs (*p* = 0.03).

About the de- and up-regulation of the genes belonging to the POLYCOMB pathway, the measured expression levels did not influence either the quality of response or EFS. Anyway, we observed a significant down-regulation of CBX2 in optimal responder patients: this gene has been reported to play an oncogenic role, with its up-regulation being able to predict progression and shorter OS in multiple cancer types (25). Thus, its reduced expression found in optimal responders could be a proof of its role also in CML. Moreover, the up-regulation of PHC3 was significantly correlated with a worse response (*p* = 0.03) and a shorter EFS (*p* = 0.025) (Figure 3).

Finally, another field of interest was the effect in term of gene de-regulation exerted by the different TKIs; nevertheless, the small number of patients receiving second-generation TKIs did not allow the observation of any TKI-specific signature.

### Principal Component Analysis Prediction Model

PCA is a multivariate analysis designed to select the linear combination of variables with higher inter-subject covariance, resulting in a good tool for gene ranking. In more detail, PCA returns a new group of orthogonal coordinates of the data space where genes are ordered in decreasing list of covariances. An experimental limitation in the present study was the difficulty in collecting a satisfying number of homogeneous patients, notwithstanding the huge number of variables (255). The principal analyzed components are usually two or three (allowing 2D or 3D plot), and the variables on each component can cluster the subjects in the matrix, thus allowing us to identify which genes would affect the principal component (and so to be significant).

**Figure 4:**
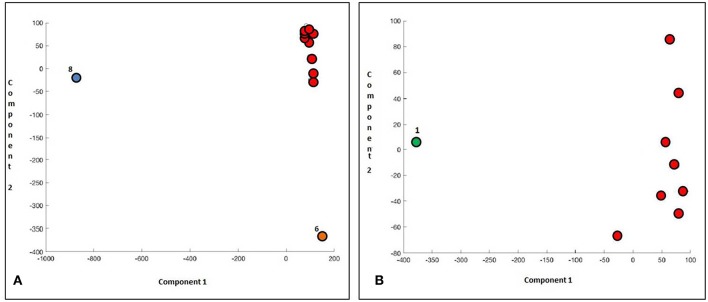
Principal Component Analysis scores represented in a 2D scatter plot. One point per sample is shown. Different colors represent separation between PCA classes. **(A)** Analysis of 255 genes in all CML patients. **(B)** Patients #8 and #6 were excluded.

Initially, we clustered in an unsupervised way the 255 tested genes and we observed that two patients stand out, respectively, for the first and the second principal component. These cases corresponded to a resistant (#8) and to a sensitive case (#6), respectively (Figure 4A). Interestingly, the genes characterizing this sample position in the matrix belonged to the WNT pathway: in particular, the first component was characterized by expression of DKK1, WNT6, DKK3, PRICKLE, WISP, and FZD8, while the second one by EP300, SFRP1, and GRB2 (Table 4).

**Table 4:** Genes scored in the principal component analysis.

**Gene**	**Median fold-change**	**Optimal**	**Failure**	**Function**
DKK1	434.25	0.25	1,302.38	WNT canonical negative regulation signaling, that acts by isolating the LRP6 co-receptor
WNT6	184.14	8.26	623.86	WNT canonical and WNT/Ca+2 non-canonical signaling.
DKK3	120.75	8.75	382.54	WNT negative regulation canonical signaling, by inhibiting LRP5/6 interaction and by forming a complex with the transmembrane protein KREMEN
PRICKLE1	111.29	3.62	363.03	WNT planar cell polarity (PCP) nuclear receptor that may be a negative regulator
WISP1	79.0	3.94	249.01	Wnt-induced secreted protein, anticancer activity, cell proliferation
FZD8	41.83	6.12	109.21	WNT seven-transmembrane domain proteins receptor activating canonical signaling
GRB2	−105.82	−159.89	4.22	STAT adapter protein growth factor receptor, activator of RAS signaling
CBX3	−113.50	−115.24	−109.14	Polycomb complex interacting gene recognizes and binds histone H3 tails methylated at Lys-9 with the E3 ubiquitin ligase Ring1B through a C-terminal domain
DNMT3B	10.65	15.19	1.55	Polycomb additional complex component DNA methyltransferase
CBX2	−22.46	−17.85	−31.66	Polycomb complex interacting gene, recruit PRC1 to chromatin by interacting with the E3 ubiquitin ligase
SFRP1	1.12	0.92	1.71	WNT canonical signaling negative regulation glycoprotein, extracellular signaling ligand
EP300	−4.99	−4.56	−5.86	WNT canonical signaling histone acetyltransferase regulating cell cycle and proliferation

*Median fold-change, median of the fold-change expression in all patients; Optimal, median of the fold-change expression only in optimal patients; Failure, median of the fold change expression in failing patients*.

In our second statistical evaluation, the two patients who previously clustered were removed, and the PCA was then applied to the remaining 9 cases: this time, a different expression profile was observed in one responsive patient (#1) who separately clustered. In this case, SFRP1 and CBX2 resulted the most significantly correlated genes: to note that the first gene belongs to the WNT pathway, and the second one to the PcGs. The values of the various components were 2.1, 0.22, and 0.16, respectively; interestingly, the plot differences were significantly related to the first component only (Figure 4B).

Then, we eliminated the case #8 and we repeated the PCA analysis: this time, two patients clustered together outside from the group: cases #6 and #1, both optimal responders. In this occasion, the latent values were 9.7, 1.9, and 0.2.

**Figure 5:**
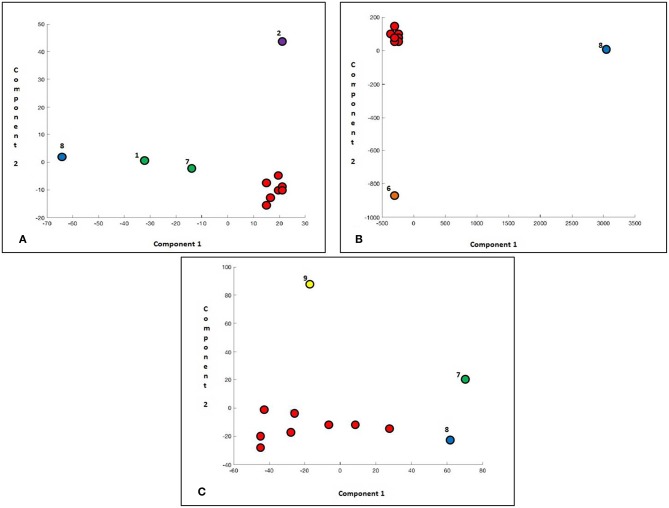
Two-dimensional scatter plot PCA scored for different pathways. **(A)** Polycomb; **(B)** WNT; and **(C)** JAK/STAT. Resistant patients #8 always scored in a PCA different class.

When the 3 pathways were separately analyzed by PCA we found that: (1) in the PcGs, the case #8 (resistant) deviated from the rest of the population predominantly on the component 1; patients #1 and #7 (optimal responders) were sited in an intermediate zone, while the remaining ones were grouped together, except for the patient #2 (suboptimal) which deviated on the component 2 (Figure 5A). The respective latent values (component 1 and 2) were: 9 and 3. The genes characterizing the component 1 were: CBX2, CBX3, and DNMT3B, while the component 2 was characterized by CBX3, HLTF, SMARCA1, PHC1, ZBTB16, and CBX2.

(2) About the WNT pathway, the main component caused a removal from the group of patient #8 whereas the case #6 differing on the component 2 (Figure 5B). The genes characterizing the component 1 were: DKK1, DKK3, WNT6, PRICKLE1, WISP1, and FZD8, while those resulting significant on the component 2 were: EP300 and SFRP1, with latent values of 1 and 0.09, respectively.

3) About the JAK-STAT pathway, two patients (#7 and #8) resulted distant on the first component, whereas #9 (sensitive) was discriminated by the second component (Figure 5C). The genes that impacted on the first component were: STAT2, IL6ST, IL10RA, FCER2, and IL2RA, whereas the genes that weighed on the second component were: SMAD3, IL6ST, and FCR2. We also found that other two genes (FCR2 and IL2RA) characterized the third component. In this analysis, the latent values were: 1.7, 1.1, and 0.8.

**Figure 6:**
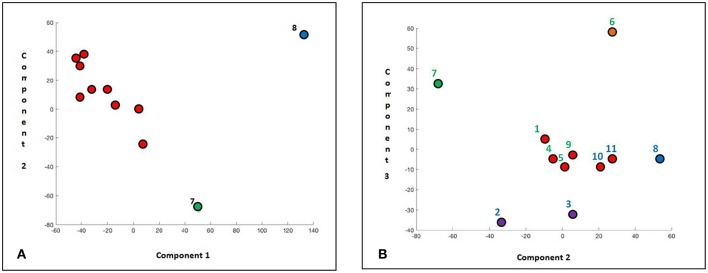
PCA 2D scatter plot of scored genes without largest fold-change. **(A)** Scatter plot on component 1 and 2. **(B)** Scatter plot on component 2 and 3.

In a second phase of our PCA analysis, a different strategy has been adopted: we removed from the analysis the genes that were over-expressed only in one patient or never expressed, obtaining latent values on the three principal components of 2.7, 1, and 0.7. Interestingly, we observed that the sample #8 (a resistant patient) clustered on the first component and that the #7 (an optimal responder) clustered on the second component, while the remaining cases were homogenously grouped (Figure 6A).

When the PCA analysis was performed only on the second and third component, the patient #7 clustered near to the zero on the second component, in opposition to the case #8 who was positioned at the opposite site of the graph (Figure 6B). On the third component, we observed patient #2 and the #3 (both suboptimal patients) at one side of the graph, and the case #6 (optimal responder) at the other side. In the center appeared #1, #4, #5 (optimal responders), and in their proximity cases #10 and #11 (suboptimal). Overall, the genes that more significantly impacted on the latest analyzed main component were: TCF7, FZD7, IFNG, SOCS3, GATA3, WNT7A, AXIN2, FZD2, GR2B, and DNMT3B, and those that conditioned the second component were FZD7, TCF7, GRB2, FZD9, FZD2, VANGL2, SOCS3, CDKN1A, CXXC4, F2R, GATA3, IL10RA, and CBX3.

A larger number of analyzed samples could permit a better score separation of samples and nevertheless our PCA analysis was able to clearly distinguish, by clustering, 2 resistant (#2 and #8) and 3 sensitive patients (#1, #6, and #7); thank to this innovative approach, we were able to detect in an unsupervised way putative genes that significantly could condition the quality of response to TKIs.

## Discussion

TKIs are widely used to treat CML, from more than 15 years, with a chance of surviving at 5 and 10 years higher than 90% (26, 27). However, TKIs fail to really cure leukemia, because the CML LSC survives and additional genetic abnormalities are believed to contribute to its resistance. Moreover, considering that only a minority of resistant cases present ABL1 mutations, the majority of causes of resistance to TKIs are probably BCR/ABL1-independent.

As above discussed, the bone marrow niche seems to have a relevant role in maintaining CML LSC: obviously, it is not easy to introduce the study of the niche in the clinical practice, so the identification of some simple laboratory approaches still represent an unmet clinical need.

In this line, some surrogate approaches have been identified, that include flow cytometry or molecular techniques: (1) the assessment by flow cytometry of the CD34+/CD38–/CD26+ cells in bone marrow but also in peripheral blood; (2) the measure of the expression level (or their change during treatment) of some genes candidate to explain the BCR/ABL1-independent resistance; (3) the evaluation of several pharmacokinetic and pharmacogenetic aspects that, influencing the TKIs plasma levels and their influx/efflux, can explain efficacy and toxicity of TKIs (drug transporters and cytochrome polymorphisms, drug interactions etc).

Because the role of CD34+/CD38–/CD26+ leukemic cells and pharmacogenetic/pharmacogenomic aspects have been already discussed in some of our previous works (7–20), in this study we focused on the gene expression profiling, in order to evaluate if de-regulation of some candidate genes at early time-points of therapy could impact on the clinical outcome.

As previously reported, gene expression profiling has been used to identify BCR/ABL1-independent mechanisms of resistance and quality of response in CML patients (28). Frank et al. in 2006 performing microarray (Affymetrix HGU133A DNA chip) gene expression analysis found that oxidative stress, DNA repair, centrosomal genes and those linked to apoptosis were associated to resistance (29).

Moreover, as demonstrated by Radich et al., deregulation of the WNT/β-catenin pathway is potentially relevant in the progression of disease. They defined gene signatures of chronic, accelerated, and blast phases comparing the gene expression in 91 CML cases (30). Of course, we do not have the same sample size, that's why our results confirmed that WNT/β- catenin involvement is a significant responsible factor of resistance more powerful than we expected.

Indeed, we compared the expression of 255 genes involved in the WNT, JAK-STAT, and POLYCOMB pathways at 6 months of treatment vs. diagnosis in peripheral blood from 11 patients, we observed that the pathway whose activation resulted more significantly correlated either with the quality of response or with the clinical outcome was the WNT/beta-CATENIN. These results have been validated in unsupervised analysis (PCA) that showed the same significantly de-regulation of WNT pathway.

The WNT/β-CATENIN pathway is known to be crucial for LSC self-renewal, perhaps because the BCR-ABL1 protein can modulate the β-catenin levels; in line with this hypothesis, in a murine model the disruption of β-catenin significantly reduced the probability of leukemic transformation (31). Interestingly, in our study, after PCA analysis, DKK1, DKK3, WNT6, and FZD8 resulted to be the most significantly de-regulated genes during treatment with TKIs. This results are perfectly in line with those already published from *in vitro* models: DKK1 and DKK3 have been shown to inhibit cancer cell proliferation (32) high levels of WNT6 inversely correlated with the response to chemotherapy in gastric cancer (33) WISP1 has been associated with a poor prognosis in glioblastoma (34, 35), and FZD8 up-regulation sustained resistance in breast cancer, prostate, and colorectal cancer (36–38).

In 2007 an Italian study demonstrated a significant gene de-regulation in the resistant patients compared to the responders ones. After a statistical analysis, the authors found a significant overexpression of 26 genes in non-responders. These genes are involved in signal transduction and transcription factors, apoptosis, cell cycle, and adhesion (39). In line with our study, the power of the proposed gene set was evaluated by two different statistical method but, to our knowledge, no studies in literature have never used both a supervised analysis and PCA at the same time in CML.

To validate results, as we decided, principal component analyses is not often used. It has been used as a biostatistical method for global approach aiming at classifying acute leukemia results of an extensive microarray study (40), in a mantle cell lymphoma study applied to a B-cell receptor-related gene signature (41), and to collect gene expression data taken from a public repository (23).

All these works and our study indicate that PCA is a feasible system that could improve validation of gene expression profiling results, by extracting significant and directly understandable information in an easy and fast way from a great amount of measures.

Translated into the clinical setting, *in vitro* studies provided several evidences on the role of WNT pathway inhibitors: Hu and colleagues used three drugs (IM, LY294002, and AKTi IV) to inhibit the BCR-ABL1/PI3K/AKT pathway in K562 cells, showing that the inhibition of these kinases induced a decrease of the β-catenin protein (but not the mRNA) and suppressed proliferation of CML progenitors (31). Zhou and colleagues demonstrated that β-catenin inhibition reverses TKI resistance in the advanced phases of disease, independently from the presence of ABL1 mutations, especially when WNT inhibitors are combined with nilotinib (42). Moreover, it has been demonstrated the involvement of the WNT/β-catenin signaling pathway in advanced phase of disease, and FZD8 seems to sustain the LSC survival (43).

WNT inhibitors (PRI-724, LGK-974, and BC-2059) are now used in advanced solid tumors, pancreatic cancer failing first-line therapy and metastatic colorectal cancer, in phase I or II clinical trial studies (https//clinicaltrial.gov), also in advanced myeloid malignancies. In our opinion, this could be a relevant item, as translated into the clinics the combination of WNT/β-catenin inhibitors and TKIs for overcoming resistance or progression in CML.

In conclusion, we found a significant de-regulation of WNT pathway and we demonstrated, even if in a small cohort of CML patients, that its activation could affect the sensitivity to TKIs and the clinical outcome.

Obviously, the aim of this study would be the possible transfer of these findings from the laboratory to the clinical real life; further studies focused on gene expression profiling analysis involving a higher number of patients could soon confirm our results.

## Ethics Statement

This study was carried out in accordance with the recommendations of Good Clinical Practice. All subjects gave written informed consent to leave leftover from the routine diagnostic samples for further no-profit studies, in accordance with the Declaration of Helsinki. The study has been performed using samples that all patients left for research purposes after signed informed consent previously approved by the Azienda Ospedaliero-Universitaria Pisana (AOUP, Pisa, Italy) Ethical Committee.

## Author Contributions

SuG, SaG, AD, and MP contributed conception and design of the study. EC, FR, SS, SB, and GE collected samples and organized databases. DL, SuG, and SaG performed statistical analysis. SaP, VM, and FGu performed gene expression analysis. ClB, GB, LI, FM, and FGh enrolled and treated patients. AC, ChB, LM, and SiP contributed to manuscript revision.

### Conflict of Interest Statement

SaG, MP, and AD received grant for speaking from Novartis, BMS, Pfizer, Incyte, MEDAC. SuG got a scholarship 2018 SIES-Incyte. All the authors declare that the research was conducted in the absence of any commercial or financial relationships that could be construed as a potential conflict of interest. The handling editor declared a shared affiliation, though no other collaboration, with one of the authors SuG.

## Abbreviations

ABCB1: ATP Binding Cassette subfamily B member 1
ABCG2: ATP Binding Cassette super-family G member 2
BCR/ABL1: Breakpoint Cluster Region/Abelson 1
BMI1: B cell-specific Moloney murine leukemia virus Integration site 1
CCyR: Complete Cytogenetic Response
CD26: Cluster of Differentiation 26
CML: Chronic Myeloid Leukemia
DKK: Dickkopf
DPPIV: dipeptidil-peptidasi IV
EFS: Event Free Survival
ELN: European Leukemia Network
EMR: Early Molecular Response
FZD: Frizzled
hOCT1: Human Organic Cation Transporter type 1
JAK/STAT: Janus Kinase/ Signal Transducer and Activator of Transcription
LabNet: Laboratory Network
LSC: Leukemic Stem Cell
MR3: Molecular Response with 3 log reduction
NGS: Next Generation Sequencing
PCA: Principal Component Analysis
PcGs: Polycomb Genes
SNP: single nucleotide polymorphism
TKI: Tyrosine Kinase Inhibitor
WISP: WNT Inducible Signaling Pathway
WNT: Wingless-related integration site.
