# Supplementary material for: The WNT Pathway Is Relevant for the BCR-ABL1-Independent Resistance in Chronic Myeloid Leukemia
Source: Front Oncol. 2019 Jun 24;9:532. doi: 10.3389/fonc.2019.00532 (PMC6601352; doi:10.3389/fonc.2019.00532)
Supplement: Supplementary file 1 [file Data_Sheet_1.docx]

Supplementary Tables:

**Table 1. WNT gene panel.**

| **FUNCTION** | **GENE** |
| --- | --- |
| **Canonical pathway** | AES (TLE/Groucho), APC, AXIN1, AXIN2, BCL9 , CSNK1A1, CSNK2A1, CTBP1, CTNNB1, CTNNBIP1 (ICAT), CXXC4, DIXDC1, DKK1, DKK3, DVL1, DVL2, EP300, FRAT1, FZD1, FZD2, FZD3, FZD4, FZD5, FZD6, FZD7, FZD8, FZD9, GSK3A, GSK3B, LEF1, LRP5, LRP6, NKD1, PORCN, PYGO1, RUVBL1, SFRP1, SFRP4, SOX17, TCF7, TCF7L1, WIF1, WNT1, WNT10A, WNT16, WNT2, WNT2B, WNT3, WNT3A, WNT4, WNT6, WNT7A, WNT7B, WNT8A. |
| **Planar Cell Polarity (PCP)** | DAAM1, DVL1, DVL2, MAPK8 (JNK1), NKD1, PRICKLE1, RHOA, RHOU, VANGL2, WNT9A. |
| **WNT/Ca+2** | FZD2, NFATC1, WNT1, WNT10A, WNT11, WNT16, WNT2, WNT2B, WNT3, WNT3A, WNT4, WNT5A, WNT5B, WNT6, WNT7A, WNT7B, WNT8A, WNT9A. |
| **WNT Signaling Negative Regulation** | APC, AXIN1, AXIN2, BTRC (bTrCP), CCND1, CTBP1, CTNNBIP1 (ICAT), CXXC4, DKK1, DKK3, FBXW11, FBXW4, FRZB (FRP-3), KREMEN1, LRP6, NLK, NKD1, SFRP1, SFRP4, SOX17, TLE1, WIF1. |
| **WNT Signaling Target Genes** | AXIN2, BTRC (bTrCP), CCND1, CCND2, DAB2, FOSL1 (FRA-1), JUN, MMP7, MYC, PITX2, PPARD, WISP1. |
| **Cell Fate** | CTNNB1, DKK1, WNT1, WNT3, WNT3A. |
| **Tissue Polarity** | AXIN2, FZD2, FZD3, FZD5, FZD6, VANGL2 |
| **Cell Growth & Proliferation** | APC, CCND1, CCND2, CTBP1, CTNNB1, CTNNBIP1 (ICAT), DAB2, EP300, FGF4, FOSL1, FOXN1, FZD3, JUN, LRP5, MMP7, MYC, PPARD, WISP1, WNT3A. |
| **Cell Migration** | APC, DKK1, LRP5, LRP6, RHOA, WNT1 |
| **Cell Cycle** | APC, BTRC (bTrCP), CCND1, CCND2, CTNNB1, EP300, FOSL1, JUN, MYC, RHOA, RHOU, RUVBL1, TCF7L1. |
| **Cellular Homeostasis** | APC, FZD2, JUN, MYC. |

**Table 2. JAK-STAT gene panel.**

| **FUNCTION** | **GENE** |
| --- | --- |
| **Janus Kinase Activity** | JAK1, JAK2, JAK3, TYK2. |
| **STAT Family** | STAT1, STAT2, STAT3, STAT4, STAT5A, STAT5B, STAT6. |
| **Regulators of the JAK/ STAT pathway** | PIAS1, PIAS2, PRL, PTPN1, PTPRC, SOCS1, SOCS2, SOCS3, SOCS4, SOCS5. |
| **Receptors that Bind and Activate JAK Proteins:** | CSF1R, EGFR, EPOR, F2R, GHR, IFNAR1, IFNGR1, IL10RA, IL2RA, IL2RG, IL4R, IL6ST, INSR, MPL, PDGFRA, SH2B1. |
| **SH3/SH2 Adaptor Protein Activity** | CRK, GRB2, SRC, STAM |
| **Transcriptional Regulation** | HMGA1, SMAD3, SPI1. |
| **RNA Polymerase II Transcription Factor Activit:** | JUNB, SP1, USF1. |
| **Transcriptional Coactivator/Corepressor** | JUNB, PIAS1, SMAD1, SMAD5, SP1, YY1. |
| **Other Transcription Factors and Regulators** | CEBPB, CRK, GATA3, IRF1, IRF9 (ISGF3G), JUN, MYC, NFKB1, NR3C1 (GRL), SMAD2, SMAD4. |
| **Genes Induced by STAT Proteins** | STAT1: CXCL9 (MIG), IRF1, JUNB, NOS2 (iNOS).  STAT3: A2M, BCL2L1 (BCL-X), CCND1, CDKN1A (p21CIP1/WAF1), CEBPD, CRP, FAS (TNFRSF6), IRF1, LRG1, MCL1, MYC, SOCS1, SOCS3. STAT4: FCGR1A, IFNG, IRF1, MYC.  STAT5: BCL2L1 (BCL-X), CCND1, CDKN1A (p21CIP1/WAF1), IL2RA, IRF1, OSM.  STAT6: FCER2, GATA3, IL4, IL4R (CD124). STAT1/STAT2/p48: ISG15 (G1P2), OAS1. |
| **Apoptosis** | AKT1, BCL2L1 (BCL-X), CDKN1A, F2, F2R, FAS (TNFRSF6), IL2RA, MCL1, NFKB1, PRLR, STAT1 |
| **Cell Cycle** | CCND1, CDKN1A (p21CIP1/WAF1), EGFR, F2, F2R, IL2RA, IRF1, JAK2, MYC, STAT1 |
| **Cell Growth** | A2M, EGFR, GHR, IFNG, IL4, INSR, OSM, SOCS1, SOCS2, SOCS3, SOCS4, SOCS5 |
| **Cell Proliferation** | CSF1R, IL2RG, IL4, MPL, OSM, PDGFRA, SMAD3. |
| **Cell Differentiation** | IL20, IL4, NOS2 (iNOS), SOCS2, SOCS5. |
| **Acute-Phase Response** | CEBPB, CRP, F2, STAT3. |
| **Humoral Immune Response** | FCER2, CSF1R, IL4, NFKB1, YY1. |
| **Inflammatory Respons** | CEBPB, CRP, CXCL9, IL20, NFKB1, NOS2 (iNOS), NR3C1 (GRL). |
| **Other Genes Involved in the Immune Response** | CXCL9 (MIG), FAS (TNFRSF6), FCGR1A, ISG15 (G1P2), IFNG, IL2RA (CD25), IL2RG, IL4R (CD124), IL6ST (GP130), IRF1, IRF9 (ISGF3G), OAS1, OSM, PRLR, PTPN11, STUB1. |

**Table 3. Polycomb and Tritorax complex gene panel.**

| **FUNCTION** | **GENE** |
| --- | --- |
| **Polycomb Complexes:** |  |
| **Core Components** | BMI1 (PCGF4), EED, EZH2, PHC1, PHC2, PHC3, RBBP4, RBBP7, RING1, RNASEL, SCMH1 |
| **Alternate Core Components** | CBX5, EZH1, PHF1, RNF2. |
| **Additional Complex Components** | CBX8, DNMT1, DNMT3A, DNMT3B, E2F6, KDM2B, KDM5D, PCGF1, PCGF2 (RNF110), PHC2, RING1, RNF2, RNF2, RYBP. |
| **Complex Assembly Cofactors** | AEBP2, RBP2, SNAI1, ZBTB16. |
| **Complex Interacting Genes** | ARID1B, ASXL1, ASXL2, ASXL3, BAP1, CBX1, CBX2, CBX3, CBX4, CBX7, CTBP2, DNMT3L, EPC1, EPC2, HTT, INO80 (INOC1), INO80B, INO80C, INO80D, JARID2, L3MBTL2, LARP7, MBTD1, KMT2B, KMT2E, MOV10, MTF2, PCGF5, PHF19, PPP1CC, PPP1R8, SCML2, SIRT1, SMARCA4, SMARCC1, TRIM27, USP11, USP7, YAF2, YY1AP1, YY2. |
| **Trithorax Complexes:** |  |
| **Core Components** | ARID1A, ASH2L, CXXC1, HLTF, KMT2D, KMT2C, PBRM1, RBBP4, RBBP5, RBBP7, SMARCA1, SMARCA2, SMARCB1, SMARCC2, WDR5. |
| **Complex Interacting Genes:** | ARID1B, KMT2B, SMARCA4, SMARCA5, SMARCC1 |

In Tables 1, 2 and 3 are reported the complete lists of the tested genes.

**Table 4. Molecular Responses.**

| **Patients** | **Timing** | | | |
| --- | --- | --- | --- | --- |
|  | 3M | 6M | 12M | Last Response |
| **1** | EMR  (0.022%) | Optimal  (0.0070%) | Optimal  (0.0058%) | Optimal |
| **2** | No EMR  (13.276%) | Optimal  (0.4611%) | Warning  (0.6824%) | Failure |
| **3** | No EMR  (41.44%) | Warning  (2.116%) | Failure  (6.2564%) | Failure |
| **4** | EMR  (0.467%) | Optimal  (0.0292%) | Optimal  (0.007%) | Optimal |
| **5** | EMR  (0.06%) | Optimal  (0.0059%) | Optimal  (0.0017%) | Optimal |
| **6** | pos | Warning | Optimal | Optimal |
| **7** | EMR  (0.928%) | Optimal  (0.337%) | Optimal  (0.025%) | Optimal |
| **8** | EMR  (7.093%) | Warning  (4.327%) | Failure  (1.883%) | Failure |
| **9** | EMR  (0.022%) | Optimal  (0.0038%) | Optimal  (0%) | Optimal |
| **10** | No EMR  (28.81%) | Failure  (63.57%) | Failure  (58%) | Failure |
| **11** | EMR  (4.49%) | Optimal  (0.3813%) | Warning  (0.2077%) | Warning |

Table 4. Molecular Responses: EMR: early molecular response; 3M: 3 month; 6M: 6 month; 12M: 12 month; pos: positive. Clinical outcome of patients in term of molecular response to TKIs, according to ELN guidelines (22).

**Table 5. I line therapy and switch of TKI.**

| **Patient** | **TKI I line** | **Switch TKI** | | | **TFR** |
| --- | --- | --- | --- | --- | --- |
|  |  | Yes/No | Why | Which |  |
| **1** | IMA | No | Optimal |  | No |
| **2** | IMA | Yes | Failure | DAS | No |
| **3** | IMA | No | Not done for comorbidity |  | No |
| **4** | DAS | Yes | Toxicity | BOS | No |
| **5** | IMA | No | Optimal |  | No |
| **6** | IMA | No | Optimal |  | No |
| **7** | NIL | No | Optimal |  | No |
| **8** | IMA | Yes | Failure | PON | No |
| **9** | NIL | No | Optimal |  | No |
| **10** | IMA | Yes | Failure | BOS/PON | No |
| **11** | IMA | No | Not done for comorbidity |  | No |

**Table 5. I line therapy and switch of TKI.**

IMA: Imatinib; DAS: Dasatinib; NIL: Nilotinib; BOS: Bosutinib; PON: Ponatinib; TFR: treatment free remission. Table of I line therapy. We have listed the TKIs resistance or intolerance that had or not caused the switch to II/ III- line of therapy.
